# Advancement in Nanostructure-Based Tissue-Engineered Biomaterials for Retinal Degenerative Diseases

**DOI:** 10.3390/biomedicines9081005

**Published:** 2021-08-13

**Authors:** Sonali Suresh Rohiwal, Zdenka Ellederová, Taras Ardan, Jiri Klima

**Affiliations:** Laboratory of Cell Regeneration and Plasticity, Institute of Animal Physiology and Genetics, The Czech Academy of Sciences, v.v.i. Rumburska 89, 277 21 Libechov, Czech Republic; ellederova@iapg.cas.cz (Z.E.); Ardan@iapg.cas.cz (T.A.); klima@iapg.cas.cz (J.K.)

**Keywords:** nanostructures, cell transplantation, retinal degenerative disease, biomaterial, implants, scaffolds

## Abstract

The review intends to overview a wide range of nanostructured natural, synthetic and biological membrane implants for tissue engineering to help in retinal degenerative diseases. Herein, we discuss the transplantation strategies and the new development of material in combination with cells such as induced pluripotent stem cells (iPSC), mature retinal cells, adult stem cells, retinal progenitors, fetal retinal cells, or retinal pigment epithelial (RPE) sheets, etc. to be delivered into the subretinal space. Retinitis pigmentosa and age-related macular degeneration (AMD) are the most common retinal diseases resulting in vision impairment or blindness by permanent loss in photoreceptor cells. Currently, there are no therapies that can repair permanent vision loss, and the available treatments can only delay the advancement of retinal degeneration. The delivery of cell-based nanostructure scaffolds has been presented to enrich cell survival and direct cell differentiation in a range of retinal degenerative models. In this review, we sum up the research findings on different types of nanostructure scaffolds/substrate or material-based implants, with or without cells, used to deliver into the subretinal space for retinal diseases. Though, clinical and pre-clinical trials are still needed for these transplants to be used as a clinical treatment method for retinal degeneration.

## 1. Introduction

**Figure 1:**
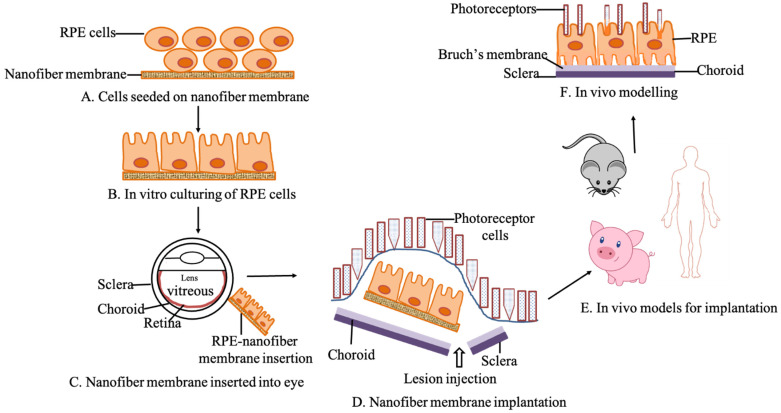
Schematic of RPE tissue engineering strategy.

The nanomaterials and nanometer-scale schemes provide superior control over the modifications in structure and function at the molecular and nanometer-scale length [1]. The challenge in interface engineering is to control the structural and physical properties of an artificial environment to mimic the biological atmosphere within the body. The retinal photoreceptors and retinal pigmented epithelium (RPE) form a functional unit and any deterioration of this association leads to the loss of photoreceptors and later leads to irreversible vision loss. To date, some therapies are present that can delay the development of retinal diseases, but there is no treatment yet that completely stops or reestablishes normal retinal function [1] and restores lost vision in these patients. The RPE monolayer replacement may exemplify a beneficial treatment option for patients with age-related macular degeneration (AMD). The RPE/choroid patch translocation and suspension RPE transplantation were attempted earlier but faced numerous technical and biological challenges [2]. However, these improvements have directed to phase I/II clinical trials in which human embryonic stem cell-derived RPE cells are transplanted in suspension subretinally for the treatment of AMD and Stargardt disease [3]. Tissue engineering provides a promising opportunity to improve cell-based therapies. The fundamental concept is to use a scaffold on which cells are pre-cultured that supports maturation into a functional monolayer and, subsequently, the transplantation of these cell-based scaffold complexes underneath the retina. Such a scaffold would facilitate a facile specific delivery and ensure the subsequent tissue-engineered scaffold compared to the prior art. For RPE monolayer transplantation, the RPE tissue-engineered scaffold should provide all the optimal conditions such as: simulate their natural microenvironment, together with the biocompatibility, suitable surgical properties and regulatory features. The scaffold’s substrate use for culturing RPE cells can be divided along with natural, synthetic and hybrid fiber formulations that have been tested for clinical and preclinical trials. These natural scaffolds mean the scaffolds whose components are derived from biological resources, whereas synthetic scaffolds mean the scaffolds whose constituents are inorganic in derivation. The hybrid scaffolds have both natural as well as synthetic elements of origin [4,5]. In many cases, the inner layers of the retina and other associated cells maintain the constructions for a while following photoreceptor loss. This recommends that if a healthy scaffold can integrate into the retina and replace the photoreceptor function, that can be an alternative to reconnect to the remaining host visual synaptic pathways and reestablish vision after delivering into the subretinal space [6]. The overall schematic representation of the RPE tissue engineering strategy is as shown in Figure 1.

Therefore, cell-based therapies can be a good alternative in combination with tissue engineering for a retinal disease that has recently advanced significantly over recent years with promising results. Several types of cell sources for cell-based therapy were projected, including adult and fetal, as well as pluripotent stem cells. Additionally, progenitor cells, mature photoreceptors, retinal sheets and RPE cells have been studied for cell transplantation to the subretinal space in animal models from 1 to 7 months [7,8]. Though, trials remain with phases, such as the delivery and integration of regenerative scaffolds to the eye, chances of immune rejection and the control of axonal growth to establish useful connections.

In the first part of the review, we focus on different type’s nanostructure-based biomaterial implants such as natural, synthetic and biological-based nanostructured material. We review and compare the characteristic differences between natural, synthetic and biological origin material. In the second part of the review, we provide data on the usage of renewable sources of donor cells for transplantation towards retinal pigment epithelium repair. To grow and characterize embryonic and fetal retinal sheets, retinal progenitor cells (RPCs), mesenchymal stem cells (MSCs), RPE replacement, mature retinal cells, adipose stem cells (ASCs), induced pluripotent stem cell (iPSC)-derived RPE on nanofiber scaffolds and discuss the relevance of nanofiber scaffolds for clinical application.

## 2. Structure and Function of Eye and Retinal Epithelium Membrane

**Figure 2:**
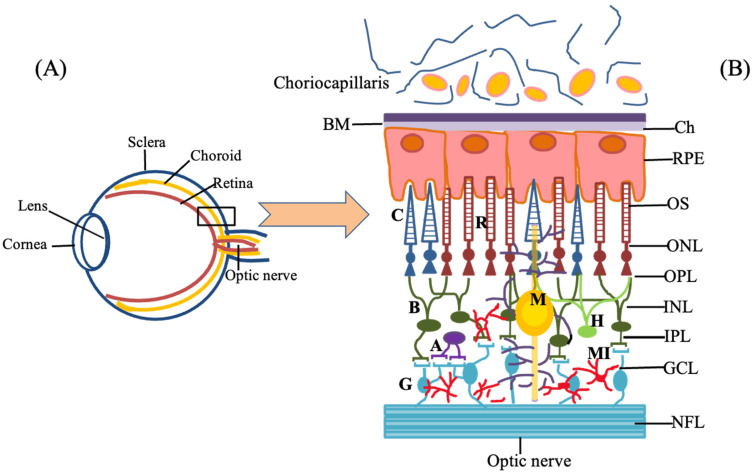
Systematic representation of (**A**) structure of an eye and (**B**) Schematic drawing of the cellular components of the retina: The different cell types are located in a mammalian retina and there are cellular interactions between the different cells such as amacrine cells (A) (in purple), bipolar cells (B) (in green), ganglion cells (G) (in turquoise), horizontal cells (H) (in bright green), Müller cells (M) (in yellow), microglia (MI) (in red), rods (R) (in dark red) and cones (C) (in blue). The figure also shows the different layers of the retina (from the most internal to the outer layers): optic nerve (ON), nerve fiber layer (NFL), ganglion cell layer (GCL), inner plexiform layer (IPL), inner nuclear layer (INL), outer plexiform layer (OPL), outer nuclear layer (ONL), outer segment layer (OS), pigment epithelium (PE), choroid (Ch), including choriocapillaris.

The human eye is a complex optic instrument organ responsible for vision by transmitting, refracting and converting the light’s energy into cellular signals for the brain to process as vision. The eye consists of different parts such as the conjunctiva, cornea, lens, retina, sclera, choroid and optic nerve as shown in Figure 2 that perform various structural and functional duties. The cornea is the clear outer part and the lens is located behind the iris which helps to focus light, or an image, on the retina. They are in charge of the refraction of light with the help of tissues, aqueous and vitreous humor through which light passes and must remain optically clear to preserve good vision. The macula is the small, sensitive area of the retina that allows central vision. It is located in the center of the retina. These engender a complex cascade of electrical and chemical events with signals transferred to the brain by the ganglion cells that encompass the optic nerve. Glial cells found in the retina (Müller glia, astrocytes and microglia) mediate transport of molecules between ganglion cells and retinal vessels and secrete neurotrophic factors. They, together with vascular endothelial cells and basal membrane, constitute the blood–retinal barrier. The glial cells perform various functions such as maintaining the brain architecture, homeostasis, nutrition and regulating neuronal communication. On the other hand, they are also involved in many other roles within the nervous system, for example, such as the maintenance of synapses, influencing synaptic connectivity by controlling the genesis, and by modifying synaptic strength and plasticity. Müller, astrocytes and microglial cells share many functions within the retina, although each has a different developmental origin. Some of these activities are performed simultaneously, while others are carried out by one or the other of the glial cell types [9].

The schematic illustration of the structure of the eye is as shown in Figure 2A. The retina is the light-sensitive tissue at the back of the eye, it transforms light into electrical impulses that are sent to the brain through the optic nerve.

The RPE is a monolayer of pigmented cells located between the neuroretina and the choroids. It is a hexanocuboidal layer of cells which is essential for the maintenance and survival of the overlying photoreceptor cells and to regulate the integrity of the choroidal capillaries. RPE cells have established a complex structural and functional polarity that permits them to perform very specific roles. RPE performs a number of critical functions, i.e., the formation of the outer blood–retinal barrier, transepithelial transport of nutrients and waste products, storage and transport of retinoids phagocytosis and degradation of spent outer segments, production of growth factors, protection against light and free radicals, visual cycle and immune response. The RPE is of neuroectodermal origin and is, thus, considered to be part of the retina. The apical membrane of the RPE faces the photoreceptor’s outer segments and its basolateral membrane faces Bruch’s membrane, which divides the RPE from the fenestrated endothelium of the choriocapillaris as shown in Figure 2B. The RPE cell membrane has discrete apical, basal and lateral surfaces, supports the tissue of the macula and is known to play a key role in retaining normal functions of the neural retina, mostly the photoreceptors. The retina and RPE are provided with blood by the central retinal artery and the choroidal blood vessels. The inner retina includes the epiretinal membrane and retinal nerve fiber layer (NFL). The middle retina includes the retinal GCL, IPL, INL and OPL. The outer retina includes the photoreceptor layer and RPE. The outer sections of these cells are structured with stacks of discs that comprise light-reactive photopigments. Due to the presence of melanin and other pigments, including lipofuscin granules which are pigmented in nature, they have a brown color that accumulates with age [10]. The visual function is mainly associated with both the INL and photoreceptors and the studies have also shown that the degree of metamorphopsia is associated with the macular thickness of the INL [11]. The cell bodies of the bipolar, amacrine and horizontal cells reside in the next layer, the inner nuclear. These bipolar cells synapse with photoreceptors in the outer plexiform layer, whereas the horizontal cells synapse laterally in the same layer. The ganglion cells collect the visual information from amacrine cells and bipolar cells in the form of chemical messages identified by receptors present on the ganglion membrane [12]. Photoreceptors are the cells that cannot regenerate after an injury such as other cells of the central nervous system due to injury or disease such as age-related macular degeneration (AMD) or retinitis pigmentosa, so there is a need to replace or regenerate these cells in such conditions [12,13,14].

## 3. Nanostructure-Based Biomaterial Implants

In recent years, many naturally occurring organic polymers such as proteins, peptides, lipids, cyclodextrins and dendrimers have been utilized as a carrier for cellular transplant approaches. Nanotechnology can direct the differentiation of stem cells, by providing growth factors as bioactive molecules that activate specific signaling pathways [15,16]. The design, synthesis and selection of an organic nanostructured material are significant steps in the development of various novel organic hybrid materials. The organic-based polymeric material provides a three-dimensional environment that can facilitate different properties viz. distinct cell adhesion, proliferation and post-transplantation migration into the host environment [17].

### 3.1. Natural Biomaterials

Natural polymers are widely used in the regenerative medicine field because of their biodegradability, biocompatibility and similarity to the extracellular matrix (ECM). These polymers combine several advantages with the ability to direct cell behavior by using materials that closely mimic the ECM of the targeted tissue type. Though, it is difficult to control the homogeneity of a naturally derived product and the mechanical properties of the resulting scaffolds. Additionally, there are limitations regarding the usage of natural scaffold materials due to the various reasons viz. purity of animal-derived polymers, the transmission of disease, mechanical instability and hypersensitivity reactions [18,19,20].

i. Gelatin

Gelatin is a natural polymer derived from collagen. It is collagen that has been processed to remove its higher-order structure. It is prepared from different animal by-products. Gelatin-based biological implants have been examined for both retinal pigment epithelium (RPE) as well as for photoreceptor transplants [21,22]. Silverman et al. (1989) observed the survival of the photoreceptor in rosette structures with the decrease in number and length of the outer segments by using 4% of gelatin scaffolds of 200–300 μm in diameter in an 8-day decrease in the number and length of the outer segments in vitro experiment on domestic cats [23]. Ghosh et al. (1999) showed that there are some surgical complications when gelatin is embedded with partial thick grafts in an ex vivo experiment on an adult embryonic rabbit. Additionally, the embryonic full-thick retina established the best integration [24]. Likewise, Hsiue et al. (2002) illustrated that a transplanted retina survived and maintained a laminar structure in rabbits, incorporated with 10% gelatin (60–70 μm thick) in a sandwich-like membrane for retinal-sheet transplantation [25]. Additionally, Khodair et al. (2003) demonstrated a retraction of photoreceptor axons as early as 10 min up to 24 h after ex vivo transplantation of adult porcine retina using gelatin sheets 250–300 μm in diameter [26]. Noorani et al. (2018) fabricated gelatin/chitosan nanofibrous scaffolds for transplantation of retinal cells. They observed that after incorporation of gelatin it enhances the electrospinability of chitosan and it also increases the ratio of degradation and proliferation owing to its hydrophilic feature. No toxic effect has been observed after cross-linking with glutaraldehyde further recommending the safe use of these nanofiber scaffolds for transferring retinal cells into subretinal space [27]. Thin-layer gelatin nanofiber mats were fabricated by Shakibaie et al. (2018); these nanofiber mats are non-toxic and biocompatible. They demonstrated that these nanofibers can be transplanted into the retina without any perturbation in nutrient diffusion. They have also demonstrated that there is a strong and effective attachment of the hRPE cells on the gelatin nanofibers [28].

ii. Collagen

**Figure 3:**
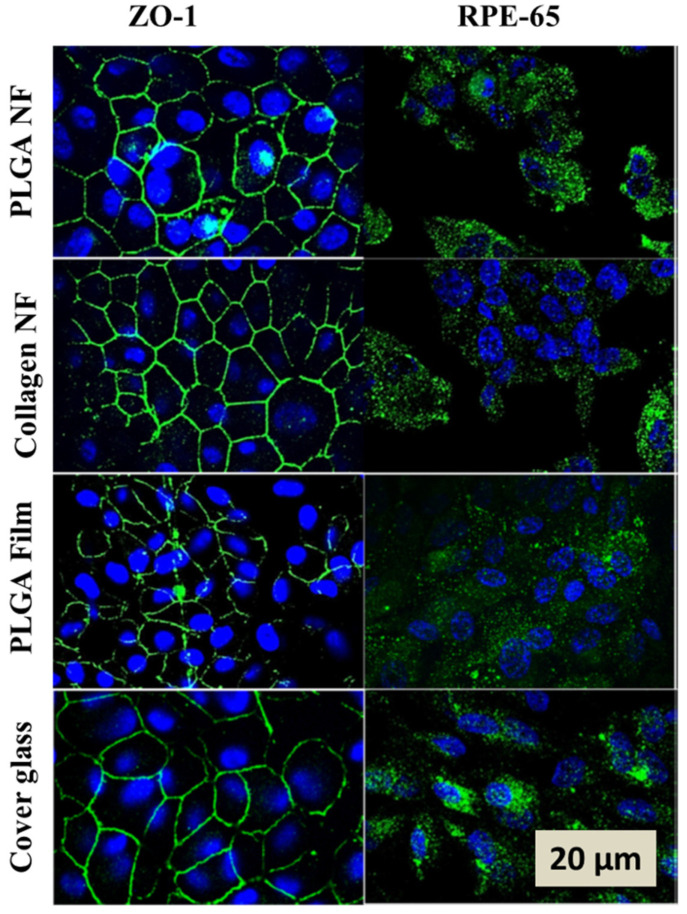
Immunofluorescence image of the expression of ZO-1 and RPE65 antibodies by human RPE cells on PLGA and collagen nanofibrous membranes. Reprinted from [34].

Collagen is the primary component of ECM presented in five different types. Wherein, types I, III, IV and V are the major components of Bruch′s membrane [18,25]. Collagen has been widely investigated as a scaffold for a variety of tissues. It can be easily cross-linked into gels of different mechanical properties and degradation rates [25,29]. Bhatt et al. (1994) carried out a study on X-linked and non-X-linked types of collagens; the result was that the non-X-linked collagen support supported RPE integrated with host RPE over X-linked collagen in albino rabbit [30]. Similarly, Thumann et al. (2006) observed that RPE cells form a monolayer with an appropriate phenotype by using non-X-linked type I collagen in albino rats [31]. Lu et al. (2007) illustrated that RPE cells form a monolayer with appropriate phenotype which could phagocytose using thin collagen film scaffolds of X-linked type I collagen on adult human RPE [32]. Additionally, Imai et al.’s (2007) grown cells on collagen type I exhibited the upregulation of angiogenic gene expression in cultured RPE cells [33]. Warnke et al. [34] compared thin films to electrospun nanofiber collagen scaffolds which showed a better cellular morphology on nanofiber scaffolds as compared to thin films (Figure 3).

iii. Fibrinogen (FG)

The cross-linked FG has been successfully used for transplantation into subretinal space by growing human fetal RPE cells in the form of microspheres. Oganesian et al. (1999) [35] observed that the cells can persist for 1 month and form a monolayer over the host retinal epithelial cells with a gentle local inflammatory response. This new xenogenic model may have significance in the study of subretinal transplant concerning cell biology and the immune response. After the incorporation of human mesenchymal stem cells (hMSCs) in FG, the maximum significant effect on cartilage marker gene expression and accumulation was observed by Ahmed et al. (in 2011) [36]. This concluded that FG is more promising than platelet-rich fibrin glue (PR-FG) as a scaffold for the chondrogenic differentiation of hMSCs. Though, the immobilization of growth factors inside these fibrin scaffolds with the heparin-based delivery system troubles this progression. Moreover, Ahmed et al. (2015) showed the use of a cell delivery system containing autologous FG which can be formed by the cryoprecipitate of fibrinogen and thrombin; consequently, the addition to form a fibrin sealant. They imagined that the provisional entrapment of retinal pigmented cells (RPCs) within the FG could stimulate better proliferation and differentiation [37].

iv. Laminin

Laminins in many cell types perform critical functions in cell attachment, growth, migration and differentiation. Laminin I is the first extracellular matrix protein to emerge during embryonic development, where it surrounds the inner cell mass of the compacted blastocyst [38]. Laminin may retain adequate conformation to stimulate the cell adhesion and growth significance of the basement membrane. Laminin is a fibrous network that self-assembles into the basement membrane. Laminin is a natural biopolymer which is used to enhance cell adhesion and penetration into the porous polymer scaffolds. Pritchard et al. (2010) synthesized electrospun nanofibers composed of laminin and PCL which shows the promotion of insufficient cell adhesion for a simultaneous transplantation of isolated photoreceptor layers and poly (glycerol sebacate) (PGS) membranes [39]. Similarly, Pritchard et al., 2010 further coated PGS membranes with electrospun nanofibers, composed of laminin and PCL, to promote the attachment of embryonic retinal explants, allowing the resulting composites to be handled surgically as a single entity [40].

### 3.2. Synthetic Biomaterials

Synthetic biomaterials have several advantages over natural biomaterials in terms of reproducibility, degradation rate, mechanical properties and shape. They provide a substitute to natural scaffolds for the culture of stem cells. Moreover, the surface properties of synthetic fibers can be easily tailored in a defined and controlled manner. Additionally, the surfaces of these modified nanofibers have shown synergistic benefits compared to seeding RPE onto nanofibers alone. The ability to modify scaffolds with a precise degradation rate is one more advantage of the synthetic scaffolds over natural biomaterials, which can also disturb the release rate of drugs encapsulated into nanofibers. However, synthetic materials lack the cell adhesion property and surface modifications may be needed to allow them to bind to specific stem cells. The biocompatibility and biodegradability are required to be considered before in vivo transplantation as its byproducts can trigger an immune response [5].

After implantation of the material, the host reactions include injury, blood-material interactions, provisional matrix formation, acute inflammation, chronic inflammation, granulation tissue development, foreign body reaction and fibrosis/fibrous capsule development. In an early process of implantation, the blood/material interaction expedites to protein adsorption to the biomaterial surface and the development of a blood-based transient provisional matrix that forms on and around the biomaterial. This further leads to thrombus formation at the tissue/material interface. The protein adsorption and fibrin-predominant provisional matrix formation are intimately linked in their mechanistic responses. This ultimately leads to the initiation of innate immunity responses formation involving the activation of the extrinsic and intrinsic coagulation systems, the complement system, the fibrinolytic system, the kinin-generating system and platelets [41].

Earlier studies have shown that nanofiber morphology may provide distinctive contact cues to regulate macrophage cells to anti-inflammatory phenotypes in vitro [42]. It was observed that the nanofibrous topography is an important regulator to decrease the foreign-body response with the indiscernible activation of macrophage cells and restrain the unnecessary macrophage activation to improve material’s biocompatibility in vivo as compared to microfibrous membranes [43].

i. Hydrogel

Hydrogels consist of a matrix of insoluble cross-linked polymers such as starch, cellulose or other animal or plant-derived polysaccharides comprised 96% of water. A hydrogel-based system for cellular transfer permits restricted delivery to the subretinal space. Ballios et al. [16] tested several hydrogels by physical properties such as physical properties such as gelation time and flow, biological properties of retinal stem-progenitor cell (RSPC) growth and cell survival to be used as an implantable material. Herein, they have formulated a hyaluronan and methylcellulose (HAMC)-based delivery system that meets the standards of being minimally invasive, injectable and biodegradable in situ. This system has been further explored and tailored for the transport of therapeutic cellular populations for the regeneration of vision in animal models of retinopathy.

ii. Poly (lactic-co-glycolic acid) (PLGA) and Poly (L-lactide-co-DL-lactide) (PDLLA)

Therapeutic angiogenesis via local delivery of protein drugs is one of the approaches to treat exudates in age-related macular degeneration. Anti-vascular endothelial growth factors (bevacizumab, ranibizumab and aflibercept) have been employed to reduce neovascularization in the eye (van Wijngaarden and Qureshi, 2008) [44]. Carrasquillo et al. (2003) prepared PLGA (50:50) microspheres comprising anti-vascular endothelial growth factor (anti-VEGF) RNA aptamer. The product was compressed after lyophilizing with and without trehalose. In these conditions, the inhibition was, in general, enhanced when it was co-lyophilized with trehalose. The microspheres (14-16 mm) released the aptamer at an average rate of 2 mg/day. The particles were loaded into a device and placed on the sclera of Dutch belted rabbits. The feasibility of delivering the bioactive anti-VEGF aptamer in a controlled manner was demonstrated [45].

Tezcaner and Hicks micro-fabricated PLGA/Poly (hydroxybutyrate-co-hydroxyvaleric acid) (P8HVB) adult porcine photoreceptors laminin and polylysine (PLL) 10 and 100 μm thick parallel groove 21 and 42 μm wide, 20 μm deep, and 10 μm apart photoreceptors demonstrated a preference for groove structures [5]. Poly (lactic acid) (PLLA)/PLGA films do provide the RPE monolayer sheet for transplantation, but in vitro cultures of human fetal RPE cells grown on these supports did not show pigmentation (melanogenesis) [46,47]. Similarly, Christiansen et al. determined the effect of membrane brightness of PLGA scaffolds on multifocal electroretinograms (mfERGs) by incorporating it into subretinal space. It was concluded that normal mfERG amplitude ratios were produced by bright subretinal objects that can do so even when the adjoining photoreceptors are absent [48]. Figure 3 shows the immunofluorescence of the expression of ZO-1 and RPE65 by human RPE cells. These cells were cultivated on the nanofibrous membranes on PLGA and collagen nano-fibrillar membranes (NF), PLGA films and cover glass after 11 days before immunofluorescence staining. These RPE cells on NF membranes form an in vivo-like monolayer. Cells on NF membranes also exhibit long, sheet-like microvilli, while cells on flat surfaces seem less organized. Popelka et al. (2015) presented the ultrathin, highly porous and surgically convenient cell carrier manufactured from PDLLA nanofibers. This carrier demonstrated the potential to improve the functionality and the integration of transplanted porcine primary RPE cells [49].

iii. Poly (methyl methacrylate) (PMMA)

Tao et al. micro-fabricated PMMA GFP+ mouse RPCs laminin and PLL non-porous and porous 6-μm thick, 11-μm pores porous scaffolds retained RPCS better during transplant, integrated cells expressed mature and immature markers.

On the other hand, PMMA scaffolds have also shown some limitations in terms of its biodegradability, it remains in the subretinal space permanently or until removed. This may interfere with retinal reattachment and increases the hazard of inflammatory responses and scarring [50].

iv. Polyglycerol sebacate (PGS)

PGS is a biodegradable and elastomeric recently developed polyester that has been synthesized and further investigated for its physical and chemical properties. Hence, these specific scaffolds may confer certain advantages in design characteristics for retinal repair being closer to that of native tissue [51]. PGS is mainly attractive for retinal transplantation because of its thickness. PGS scaffolds having a thickness of around 45 μm with surface modification have mechanical properties that can facilitate surgical implantation. Niklason et al. (1999) successfully loaded seeded PGS grafts (with donor cells) into the preferred area by loading the graft into a syringe followed by unrolling into the subretinal space [52]. Additionally, PGS scaffolds are biodegradable. These scaffolds have an ideal degradation profile without swelling, without the loss of mechanical strength relative to its mass and it aids in the retention of the geometry of scaffolds compared to PLGA. PGS scaffolds showed high levels of cell survival, adherence and proliferation and have proved to be suitable for progenitor cell cultures. PGS scaffolds alone can induce mouse retinal progenitor cell differentiation; generate functional neurons [53]. As compared to PLLA/PLGA, PGS is a soft and elastic material having an elastic modulus of 1.66 ± 0.23 MPa while PLLA/PLGA is brittle having an elastic modulus of 9.0 ± 1.7 MPa. The maximum strain at failure for PGS was obtained to be 113 ± 22%, whereas for PLLA/PLGA it is 9%. A PGS scaffold possesses enhanced mechanical properties alike to those of retinal tissue [54]. Cells that travelled into the host retina were observed after loading PGS grafts with cells surgically in the subretinal space of C57bl/6 or rhodopsin knockout mice [55].

v. Poly-urethanes

Poly-urethanes are biodegradable, soft elastomer polymers which can be engineered into a desired shape and size. The polymer has block copolymers with a hard and soft segment, the properties can be manipulated by changing the ratio of the components [56]. The biostability of polyurethanes have been assessed by several researchers especially for its in vivo use. They found that polyurethanes are susceptible to oxidative degradation particularly in stressed environments. Thus, the biostability and mechanical properties of polyurethanes could be designed to be appropriate for retinal implants. The polymer can be cast into thin films and synthesized by electrospinning to create a porous substrate [57]. Williams et al., 2005 showed that air plasma treatment, after incubation in distilled water of pellethane and Tecoflex, significantly enhances the adhesion and monolayer formation of ARPE-19 cells. This study also demonstrates that the selection of polyurethane or surface modification can enhance the RPE response. Silva et al., 2011 attempted to transplant RPE cells on the biodegradable supports of polyurethane films. These polyurethane films exhibit suitable mechanical properties for trans-scleral-driven subretinal implantation and could be considered as supports for a functional ARPE-19 monolayer [58]. Similarly, in 2013, Silva et al. synthesized a montmorillonite clay-based polyurethane nanocomposite having biodegradable and biocompatible properties. This nanocomposite was able to interact with ARPE-19 cells providing better adhesion, migration and proliferation, essential in forming an RPE monolayer. Moreover, the nanocomposite did not elicit any immune response or hinder the surrounding ocular tissues, particularly the retina. However, one of the disadvantages of using polyurethanes is their hydrophobic surface, which is not well suited for growing a monolayer, because of poor cell adherence and spreading of the cells [59].

vi. Thermo-responsive polymer

von Recum et al. 1998 utilized thermoresponsive polymers: N-isopropyl acrylamide (NIPAAm) and 4-(N-cinnamoylcarbamide) methyl styrene porous tissue culture inserts. It was observed that the cultures of the RPE were able to reestablish an environment similar to in vivo by establishing a tight junction barrier membrane upon confluence. RPE cells formed a monolayer with an appropriate phenotype [60]. Additionally, Fitzpatrick et al., 2010 had two thermoresponsive, bioactive cell scaffolds with the backbone of bovine collagen type I with linear chains of poly (-N-isopropyl acrylamide-) (PNIPAAm). These scaffolds showed rapid and sub physiological phase transition temperatures, which aid in noninvasively delivering a liquid suspension of cells creating a cell-loaded scaffold. The researcher showed the successful use of PNIPAAm-grafted collagen as a carrier for the distribution of therapeutic cells into the subretinal space [61].

vii. Poly (e-caprolactone) (PCL)

PCL is a biodegradable polymer with a slow degradation rate (usually over longer than 3 years), it produces degradation products that are comparatively less acidic [62,63]. Recent studies using PCL as a substrate for retinal progenitor cell culture and transplantation have had promising results. So far, PCL scaffolds are the thinnest biodegradable substrates about 5 μm in diameter, utilized in retinal tissue engineering. PCL scaffolds are highly permeable allowing the passage of physiologically crucial molecules and can be positioned into the subretinal space with minimum physical distortion [64]. Redenti et al., 2008 showed that the structured PCL scaffolds improved the expression of mature photoreceptors and bipolar markers and that the PCL-delivered RPCs migrated into both degenerative and normal retina [65]. Chen et al., 2011 synthesized nanofibrous scaffolds of chitosan-graft-poly (ε-caprolactone)/poly (ε-caprolactone) having a diameter of 656 ± 53 to 925 ± 42 nm. The chitosan-PCL/PCL nanofibrous scaffold favored the proliferation and differentiation of mouse retinal progenitor cells (mRPCs) towards neuronal lineage. These scaffolds were highly hydrophilic due to the presence of surface amino groups [66]. Similarly, Peng et al., 2013 [67] reported balloon-expandable self-locking PCL biodegradable stents having 6 and 8 mm in diameter for retinal detachment. The PCL stents could be a perfect replacement for non-absorbable scleral buckling materials [67]. Christiansen et al., 2012 developed and implanted PCL scaffolds subretinally in porcine eyes. These scaffolds caused no inflammation with limited tissue disruption. These PCL short nanowires had the most proper degree of stiffness for surgical delivery into the subretinal space [65].

Liu et al., 2014 developed polyethylene terephthalate or PCL scaffolds of 200–1000 nm in diameter to be delivered subretinally into rabbits with human fetal RPE cells. The results signified that RPE 200 nm scaffolds exhibited the highest cell densities. The adherent monolayers attained a deeper pigmentation with further uniform hexagonal tight junctions [68]. Likewise, McHugh et al. 2014 determined the in vitro effect of cell culture substrate properties on RPE maturation, gene expression and function. Their study indicated that a porous scaffold allows a trans-scaffold metabolite transport and advances the RPE cell behavior as likened to nonporous PCL or porous polyester trans wells [69]. Thin, porous poly (L-lactide-co-caprolactone) (PLCL) membrane has been created by Sorkio et al., 2014 that could allow the development of a functional hESC-RPE monolayer in serum-free culture conditions. With the help of atmospheric pressure plasma processing and coating it with collagen IV, the membranes were modified to enrich cell growth and maturation [70]. The effects of vitronectin-mimicking oligopeptides incorporated in PCL films have been studied by Lawley et al., 2014 on human RPCs. The incorporation of vitronectin-mimicking oligopeptide into PCL increases cell adhesion and has shown to be favorable for driving cell differentiation [71]. Recently, Shahmoradi et al., 2017 introduced a low diameter (185.8 nm), high porosity (82%) and appropriate hydrophilicity (with contact angle 7.48 degrees) culture substrate which can be potentially used for hRPE cell transplantation. These scaffolds showed more biocompatibility, water adsorption, wettability, cell viability and cell adhesion properties which is an important factor for retinal tissue engineering. Furthermore, to utilize any of these scaffolds, functional tests such as phagocytosis should be carried out and also an in vivo experiment is crucial before human trials [72].

Biocompatibility (or tissue compatibility) is the ability of a material to perform the correct function in the body with an appropriate host response after its application and without having any toxic or injurious effects on biological systems. It is a summary of the biological, physical (mainly mechanical) and chemical properties of material important for the surviving host or transplanted cells or tissues. Many studies in the past have demonstrated various types of biomaterials with a high grade of biocompatibility. Especially natural biomaterials such as collagen or laminin are indeed very well tolerated in the body and seemed to be very suitable for direct contact with host tissues. Unfortunately, these materials very often had many disadvantages such as low chemical stability quite often associated with a very short time of degradation in the body, unstable mechanical properties complicating material delivery into the eye, bad structure with very low porosity or a very low diameter of pores hampered transportation of macromolecular substances among the cells, including nutrients or waste products. Therefore, recently, many natural biomaterials have found employment more likely in the improvement of the surface of artificial polymeric scaffolds by coating technique. The role of artificial biocompatible biomaterials is growing with the rapid development of new chemical substances. These materials have exactly determined properties with respect to biocompatibility and the high success rate of the implantation procedure. Recently, the artificial scaffolds mimicking Bruch’s membrane were developed for RPE replacement (Popelka et al., 2015) [49]. These scaffolds have a high porosity (around 80%), low thickness (10 μm), and adequate rigidity for maintaining the plain shape during implantation surgery. These characteristics were reached by the use of a poly-lactic nanofibrous membrane where the extremely high porosity with large 3D pores (interconnected vertical and horizontal pores preventing clogging) enabled perfect a simulation of Bruch´s membrane properties. Table 1 demonstrates the important information about forms, advantages and drawbacks and applications of the most used biomaterials for implantation purposes.

viii. Other Polymers

Several other polymers, including methacrylate/methacrylamide, Polydimethylsiloxane (PDMS), cryo-precipitate, RPE extracellular matrix (ECM), polyimide and micro-photodiode array materials, have also been explored by the researcher for transplantation. The effect of poly (2-hydroxyethyl methacrylate) (Poly HEMA) substrate was studied by Nazemroaya et al., 2017 on the growth, differentiation and plasticity of human RPE cells (hRPE). hRPE cells were isolated from neonatal human globes and grown on the poly HEMA) substrates. This hydrophobic polymer appears to be auspicious for both the maintenance and de-differentiation of hRPE cells and the development to the cell cultures bearing retinal progenitor cells [73]. Another clinically approved widely used polymer that owns the physical properties essential for a transplanting device is PDMS. The cellular behavior was studied by Lim et al., 2004 of cultured RPE cells affected by the manipulation of early focal contact. The HRPE and ARPE-19 cells were seeded onto the fibronectin-coated circular pillar PDMS surfaces of 5 µm diameter [74]. The properties of PDMS were manipulated by Krishna et al., 2007 so that it could support an intact functional monolayer of RPE cells. Additionally, RPE monolayers were able to exhibit the phagocytosis of photoreceptor outer segments in a time-dependent manner similar to control [75].

The polymer that shows a significant role in the development and regeneration of various cells and helps in the inhibition of RPE apoptosis is ECM. Ho et al., 1997 established a technique to collect and transfer native ECM formed by bovine, porcine and human cell lines. The potential of cultured RPE cells to reattach the harvested ECM is having the ability to inhibit apoptosis and promote survival of the transplant [22]. Similarly, human RPE cells were collected for transplantation which may lead to the separation of the cells from their extracellular matrix and induce apoptosis. Tezel et al., 1997 examined the reattachment of RPE to a substrate decreasing the rate of RPE apoptosis during in vitro analysis [76]. On the structure and function of human embryonic stem cell-derived retinal pigment epithelial (hESC-RPE) cells, the role of ECM proteins was investigated by Sorkio et al., 2014. They showed that the cell culture substrate has a foremost effect on the structure and basal lamina production during differentiation and maturation. The hESC-RPE potentially influences the achievement of cell survival and integrations after the cell transplantation process [70].

Yet, another biomaterial used for clinical purposes is cryoprecipitate especially used for the treatment of bleeding disorders or as tissue glue in surgical patients. It comprises significant amounts of fibrinogen and fibronectin. Cryoprecipitate is formed when the precipitate of fresh frozen plasma (FFP) is thawed at 4 °C and then refrozen in plasma (stored for up to a year). Siar et al., 1999 prepared thin membranes isolated from sheets of human cryoprecipitate which were attached to the membranes. These membranes may offer an ideal source for the adhesion, cultivation and transfer of human fetal retinal pigment epithelial cells for their application as a carrier into subretinal space [77].

Several retinal disorders lead progressively to the deterioration of photoreceptors and blindness. The idea of reestablishing vision to patients with various therapeutic concepts such as visual prostheses is an attractive treatment method. Some researchers implanted a micro photodiode array (MPDA) directly into the subretinal space to change the deteriorated photoreceptors. Subretinal MPDA is a type of visual prosthesis utilized for embedding in the subretinal space of patients with escalating photoreceptor cell loss. Guenther et al., 1999 evaluated cell survival and cell adhesion of mammalian retinal neurons on various materials used for the manufacture of multi-photodiode arrays (MPDAs) proposed for implantation in the subretinal space of patients suffering from advanced photoreceptor cell loss. It was found that most of the materials were suitable for the production of functional MPDAs which were later implanted into subretinal space without any complications [78]. Hui et al., 2007 also presented the use of MPDA without releasing any harmful substance or cellular toxicity which will be a barrier for in vitro RPE function. They also evaluated the effect of materials for MPDA on the apoptosis and cell viability of the cultured RPE cells. The material showed no difference as compared to the controlled group which exhibited a good biocompatibility [79].

**Figure 4:**
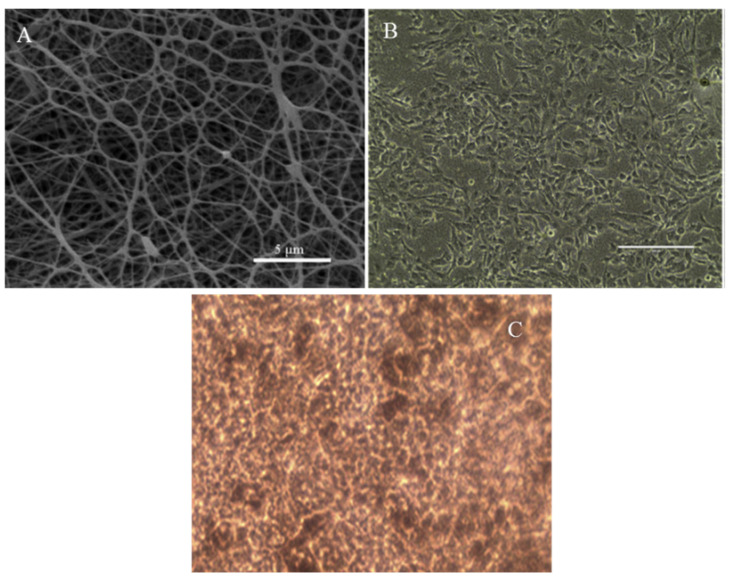
(**A**) SEM micrographs of PCL/RWSF/Gt. Scale bar = 100 µm, (**B**) ARPE-19 cultured on PCL/RWSF/Gt membranes for 24 h and (**C**) establishment of long-term cultures of ARPE-19 cells on PCL/RWSF/Gt membranes in which ARPE-19 cells displayed much tighter junctions, smaller volume and melanin pigment appears in cells on 90th day. Reprinted from [80].

Silicon rubber (PDMS) has been extensively used in medical devices where its hydrophobic qualities have been exploited in detail. The cellular behavior of RPE-cultured cells was influenced by the management of early focal contact. The micro patterned surfaces were implemented by microfabrication to control the early focal contact and reduce the focal cell-substrate contact area on PDMS substrate. Although, no substantial influence on cell adhesion was found, the major inhibition of cell cycle progression was observed statistically for micropatterned PDMS surfaces by Jung et al., 2004 [80]. The PDMS discs were used as a promising surface for the growth of healthy RPE monolayers, yet the delivery of isolated cells can cause serious usages to develop a suitable transplant membrane that could support an intact functioning RPE monolayer (Krishna et al., 2006) [75]. Xiang and his group focused on the preparation of electrospun scaffolds which has properties such as thickness and high porosity similar to Bruch’s membrane fibers made from Antheraea pernyi silk fibroin, PCL and gelatin. These scaffolds mimic Bruch’s membrane and are suitable and sustainable for the long-term growth and culture of functional RPE cells. These scaffolds accelerate RPE cell growth and proliferation without any inflammation reaction, further promoting the functionalization of RPE cells which may lead to become an applicable carrier for RPE transplantation [4] as shown in Figure 4.

## 4. Biological Membrane-Based Implants

Biological membrane-based implants possess the advantage of having a natural structure [81,82]. These nanofiber membranes closely match with the natural physiological properties of these natural membranes such as mechanical properties, the concentration of protein, morphological properties, non-immunogenicity and biocompatibility. Different types of the human membrane such as Descemet’s membrane, amniotic membrane, Bruch’s membrane and inner limiting membrane have been utilized for further development.

### 4.1. Amniotic Membrane (AM)

The human amniotic membrane (hAM) is the inner membrane enwrapping the fetus in the amniotic cavity which is thin and elastic. It consists of an epithelium membrane on a thick basement membrane, which separates it from the underlying avascular stroma. This stromal matrix has been successfully used for surface restoration in several ocular surface disorders such as ocular cicatricial pemphigoid, severe pterygium and chemical burn and Stevens–Johnson syndrome. It has been shown that the amniotic membrane serves as an adequate substrate for in vitro growth and expansion of conjunctival epithelial progenitor cells, revealing the use of AM for the treatment of acute ocular disorders [83]. Capeans et al. (2003) studied the adherence and growth of hRPE cells over hAM constituted by tight colonies that retained epithelial phenotype which ultimately showed cell–cell integration, making them suitable substrate for human retinal pigment epithelial cell (hRPE) growth [84]. Grueterich et al. unfolded the usage of AM as a basement membrane-containing matrix to maintain RPE phenotype in vitro and may facilitate and suggest transplantation to treat age-related macular degeneration (It also suggests the use of constitutes that act as a suitable substrate for in vitro growth and the expansion of conjunctival epithelial progenitor cells) [83]. Furthermore, Shimazaki et al. studied the effect of cultivated human limbal epithelium on AM for the treatment of severe ocular disorders after caring out short-term clinical trials [85]. Earlier studies by Rosenfeld et al. elucidate the use of rabbit models to demonstrate the use of biological-based subretinal space without any inflammation [86]. Furthermore, Kiilgaard et al. investigated that after the transplantation of AM into the subretinal space there is regrowth of RPE cells which prevents choroidal neovascularization membrane after basal membrane (BM) damage which aids as a BM substitute for the RPE [87]. All these results confirm that biological-based membrane, i.e., AM, is ideal for implantation into the subretinal space for retinal therapy.

### 4.2. Bruch′s Membrane Fibers

Bruch’s membrane is a membrane with collagen woven fibers that is present beneath the retina and choroid and functions as a molecular sieve to control the mutual exchange of fluids, nutrients, oxygen and waste material to a degree. This membrane acts as a support for the functional cell, and the sheet formation also functions as the substratum of RPE and vessel wall. It is a five-layered extracellular matrix, thin around 2–4 µm in diameter between the retina and choroid. It has clinical importance in AMD and other retinal diseases. It has been anticipated by researchers that RPE cells can be seeded on a biocompatible and biodegradable prosthetic BM for use in cell transplants [88]. Sugino et al. emphasized classifying the bioactive components of a bovine corneal endothelial cell-extracellular matrix so an improved clinically appropriate product can be developed and deployed to increase the transplant success in patients. It was observed that survival and differentiation of human embryonic stem cell-derived retinal pigment epithelium (hES-RPE) and human fetal RPE on aged or AMD Bruch’s membrane were boosted significantly in the presence of a biologically synthesized bovine corneal endothelial cell-extracellular matrix. This is an approach to protect the damage of Bruch’s membrane in AMD by elaborating their own ECM if it’s laid down on Bruch’s membrane before transplantation. The transplanted RPE survival is improved to 400–1000% if Bruch’s membrane is treated with this special medium through organ culture of hES-RPE or fetal RPE on aged or AMD Bruch’s membrane [89]. Alternatively, Moreira et al. studied the effects of BM reengineering on RPE phagocytosis. They previously studied that BM-derived from the eyes of older individuals reduces the ability of the RPE cultured on its surface to phagocytize ROS in vitro. Additionally, they reported the effect of cleaning the old donor-derived BM with detergent and recoating them with ECM ligands that eventually increase the survival rate, proliferation and attachment of the RPE cells [90]. Other studies showed the prevention of photoreceptors loss by the debridement of Bruch’s membrane after the transplantation of autologous RPE cells led to a repopulation of the cleaned area of Bruch’s membrane [91]. Moreover, to age-related changes to Bruch’s membrane that can at the minimum be held responsible for a failure of survival of grafted RPE cells, mechanical destruction due to the transplantation itself may result in poorer adhesion of the graft. The basement membrane of RPE is one of the most suitable for transplanted RPE cells and its abstraction causes severe consequences for RPE existence which has been proved in ex vivo studies [92]. All these studies, therefore, suggest that the Bruch’s membrane is a potential natural form to be used to treat AMD diseases.

### 4.3. Lens Capsule

**Figure 5:**
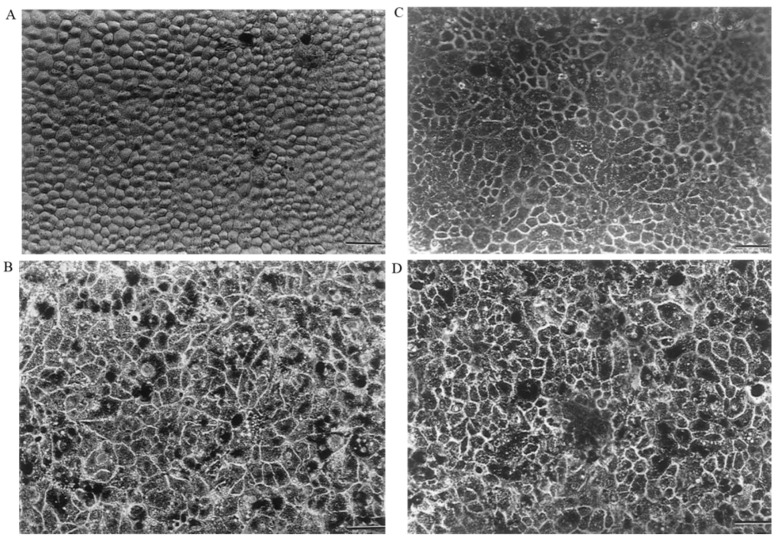
(**A**) Pig RPE cells were cultured on a pig lens capsule (100x), scale bar = 50 µm; (**B**) human RPE cells on a human lens capsule (100×), scale bar = 50 µm; (**C**) pig RPE cells cultured on a hydrogel (100×), scale bar = 50 µm; (**D**) human RPE cells cultured on a hydrogel (100×), scale bar = 50 mm. Reprinted from [80].

A Lens capsule can be a suitable substrate for the growing and transplantation of cultured monolayers of RPE and iris pigment epithelial (IPE) cells into the subretinal space and anterior lens capsule. Hartmann et al., 1999 formed stable monolayers of IPE cells on anterior lens capsules and transferred them to secondary culture flasks without causing damage to the cells [93]. For transplanted porcine RPE cells, Nicolini et al., 2000 investigated the usage of the ocular basement membrane as a substrate, but RPE cells grown on a porcine anterior lens capsule and ECM obtained a better morphology [94]. Similarly, Christina J. et al., 2002 fabricated a lens capsule by micro-contact printing a modern fabrication technique. This technique was further used to engineer the surface of human tissue. The RPE cells and IPE cells cultured onto an untreated lens capsule exhibited a dispersion and formed into fusiform-appearing cells [95]. Similarly, in 2007, Christina J. et al. replaced Bruch’s membrane with a human anterior lens capsule and investigated different seeding methods inducing the desired cellular function and morphology. The enhancement with a 1.5-fold increase in metabolic activity, over conventional gravity seeding and patterning, was also observed [96]. Similarly, Singh et al. successfully cultured porcine and human RPE cells on lens capsules and hydrogel. The cells were proliferated within 24 h and confluent monolayers were formed of polygonal epithelioid cells over the next 4–5 days (Figure 5). All these findings suggest that the lens capsule is an appropriate candidate to be used as a transplantable material [80].

## 5. Renewable Sources of Donor Cells for Transplantation towards Retinal Pigment Epithelium Repair

In the past few decades, great progress has been witnessed in the field of tissue engineering. The fundamental principle is that the dissociated cells have the propensity to reassemble into structures that restore the original tissue. To direct and control cell behavior, a tailored biomimetic environment that surrounds the cell and promotes specific cell interactions is necessary. Therefore, by manipulating the critical environmental parameters of the scaffolding material and the external physical stimulations, it is possible to provide the same microenvironment. The three-dimensional artificial nanofiber membrane/scaffold is, thus, designed to not only provide the initial structural integrity for cells, but also to direct the cell differentiation and proliferation, eventually leading to the assembly of functional tissue. The characteristic factors such as the mechanical, chemical and physical properties of the nanofiber membrane play a vital role in adapting the functional behavior of tissue regeneration. Therefore, there is a need to develop a material that acts as a substrate for cell proliferation and growth, ultimately leading to the development of implantable material.

Delivering the cells to the targeted site in the case of degenerative diseases is an attractive treatment option. The cell replacement therapy that leads to a replacement of diseased cells by healthy cells is particularly intriguing for degenerative eye diseases that involve the use of different types of cells. The most essential requirements for the implanted cells are to be compatible with the host tissue, non-immunogenic and should survive within the subretinal space. The main property of cell therapy is that it should be capable of functionally renewing the cell population that has undergone deterioration. Additionally, it should also provide enough trophic support to promote host cell survival and aid in the early stages of the disease. Moreover, the regeneration of these cell layers would require transplanted cells to be functional after implantation into subretinal space [97,98,99]. These cells may regenerate the host visual synaptic pathways and restore vision after integrating into the subretinal space of the retina and replace photoreceptor function.

**Figure 6:**
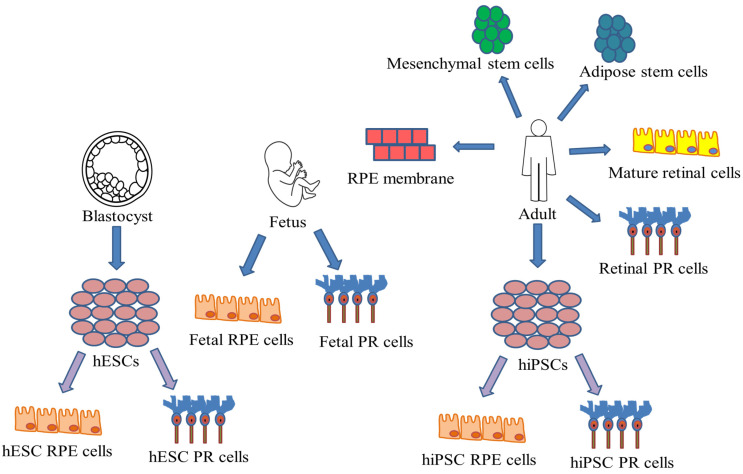
Schematic representation of cell sources for the cell therapy of retinal degenerative diseases.

Furthermore, cells must reinstate the deteriorated RPE layer with an organized monolayer of cells that can help photoreceptor endurance and maintenance. Different types of cell populations have been investigated for cell delivery into the subretinal space that can accomplish these transplantations processes. Six main types of cells can be utilized for retinal regeneration: embryonic and fetal retinal sheets, mature retinal cells, progenitor and stem cells, homologous and autologous transplants, mesenchymal stem cells and adipose stem cells. Figure 6 represents several cell sources that could be used for the cell therapy of the eye. Many cells of these groups have been effectively transplanted into animal models and some therapies are even instigating into Phase I/II clinical trials [100].

### 5.1. Embryonic and Fetal Retinal Sheets

Embryonic stem cells (ESCs) are self-renewing; pluripotent cells, which grow indefinitely in optimum culture conditions [101]. Several researchers have found the possibility to utilize the ESCs for transplantation into the retina by reprogramming them into photoreceptors or retinal ganglion cells (RGCs) [101,102]. Additionally, Lamba et al., 2009 observed that ESC-derived photoreceptors are also able to restore some level of visual function in Crx-deficient mice [103]. ESCs were also utilized in retinal diseases for example in age-related macular degeneration (AMD) and Stargardt Macular Dystrophy. The first phase I/II clinical trials was carried out in 2011, testing the safety of hESC-derived retinal cells to treat patients of retinal diseases. The hESC-derived RPE cells exhibited no symptoms of tumor genesis, hyper-proliferation, ectopic tissue formation or evident rejection after 4 months. Thus, the safety evidence for medium-term to long-term follow-up was proved [87]. So far, there are challenges with ESC derived cell transplantation which comprises the ability to acquire stable cell sources that integrate safely into the diseased retina. Moreover, there is also a risk that the plasticity of ESCs may be difficult, raising the risk of inappropriate progeny such as tumors [104,105]. Secondly, there is a challenge to develop a realistic up-scaling. The third is to generate a high number of cells required for transplantation into patients [105]. The third problem is the risk of immune rejection. Retinal degeneration results in the upregulation of inflammatory proteins and alteration of the blood–retinal barrier and this can increase the chance of immune rejection. Thus, a better understanding of an immune system is required for degenerated retinal diseases [106]. The fourth problem deals with the successful integration of stem cell-derived retinal cells viz. RGCs and photoreceptors into the retina during transplantation procedures [107]. Researchers have reported progress in visual acuity in patients by delivering retinal tissue with its associated RPE layer [108,109]. However, difficulties are associated with the availability of donors as well as with isolating and implanting the intact tissues [110,111].

### 5.2. Retinal Progenitor Cells (RPCs)

RPCs are obtained from donated fetal tissue, not embryonic tissue; therefore, they are characteristically more predisposed towards a cell fate in comparison to stem cells [112]. Klassen et al., 2004 successfully transplanted RPCs into animal models of retinal degenerative disease [101]. Similarly, Luo et al., 2014 showed an improved visual acuity in rats due to photoreceptor preservation via transplanted RPCs [113]. For retinitis pigmentosa, the phase I/II clinical trials for patients are currently common in the shown company named ‘ReNeuron’ [114,115]. The main challenge for successful transplantation into patients is the integration of cells into the complex human retinal circuitry. Afterwards, the ultimate challenge will then be whether the photoreceptors can function well enough to improve visual acuity if evidence for photoreceptor preservation is observed. Moreover, there could be a chance of immune rejection if donor tissue is used to obtain RPCs such as ESCs. Neural progenitor cells (NPCs) [116] have an established extensive integration in the developing and degenerate retina and exhibit the morphologies of all major cell types of the retina [117,118]. NPCs survive up to 18 weeks post-transplantation and differentiate into neuronal phenotypes in the Royal College of Surgeons (RCS) rat model of retinal degeneration disease [118]. Similar results were obtained using mouse whole brain-derived NPCs by Mizumoto et al., 2003 [119]. NPCs can replace different cells such as photoreceptors, neurons and glial cells. Glial cells are thought to protect neurons from various neurological insults. Müller glia and astroglia play a crucial role in the development of retinal disorders such as glaucoma. Müller cells which are the predominant glial element in the retina, undergo significant morphological, cellular and molecular changes when there is an injury to the retina. Some of these changes reflect Müller cell involvement in shielding the retina from further damage. Müller cells express neurotransmitter transporters, growth factors and antioxidant agents that could have an important role in preventing excitotoxic damage to retinal neurons. They also contact endothelial cells to accelerate the neovascularization process during hypoxic conditions. Recent studies have also observed the role of Müller cells in retina regeneration after damage, dedifferentiating to progenitor cells and then giving rise to different neuronal cell types [120]. With a potential for self-renewal, the ciliary body and iris pigment progenitor cells contain a mitotically quiescent population of neural progenitors that reproduce to make neural stem cells. Researchers also demonstrated that functional integration is possible only in damaged tissues after the incorporation of these cells into the injured retina, but not into the normal one [121]. In conclusion, these cells can differentiate into photoreceptors in vivo and hold great promise for replacing damaged photoreceptors when they were isolated from mouse retinas during the timeframe corresponding to photoreceptor development [122,123,124].

### 5.3. Mesenchymal Stem Cells (MSCs)

MSCs are multipotent stem cells derived from mesenchymal tissues such as the umbilical cord, placenta, bone marrow and adipose tissue [112]. MSCs have been found to separate into RGC and photoreceptor-like cells for the future possibility of cell replacement therapy. MSCs also aid to provide neuroprotection for degenerating retinal cells using an expression of a range of neurotrophins beneficial for retinal cell survival [125]. Researchers have also reported that MSCs can be brought in vitro into cells expressing photoreceptor lineage-specific markers using taurine, activin A and Epidermal Growth Factor [126]. Additionally, when injected into subretinal space MSCs slow down the cell degeneration process and differentiates into photoreceptors in RCS rats [127]. Moreover, Anna et al. in 2013 demonstrated that after the injection of MSCs intravitreally into a mouse model of acute retinal injury, the transplanted cells survived for at least 3 months and protected damaged retinal cells [128].

### 5.4. RPE Replacement

Replacement of the RPE is another possible way of cell replacement therapy. This cell type is an ideal candidate for transplantation because of its capability to generate large amounts of functional RPE from ESCs and iPSCs [129]. RPE transplantation in suspension and RPE/choroid patch translocation have previously faced several challenges such as the eventual rejection or a lack of functional recovery, diminished cellular structures and the slowing of metabolic capacity triggered by an accumulation of lipofuscin due to the process of ageing and degenerative disease. Schwartz et al., 2012 transplanted ESC-derived RPE into patients with Stargardt macular dystrophy and AMD. They observed that transplanted RPE had no seeming rejection after 4 months. Hence, the challenge of immune rejection could be overcome and potentially used for photoreceptor and central visual rescue if further developed [130].

RPE cells are not homogeneous throughout the retinal monolayer. Yet, the best approach would be the transplantation of RPE cells from the periphery of the recipient RPE monolayer [131]. Another approach to avoid graft rejection is the autologous transplantation of iris pigment epithelium (IPE). Through an iridectomy, autologous IPE cells can be easily acquired and may be frequently isolated from the donor or host [132]. To study the integration and function of peripheral RPE alongside the existing foveal RPE, additional information is needed.

Many researchers have reviewed the transplantation strategies for RPE cells [133] RPE cells suspensions were isolated and transplanted into the subretinal space of either allogenic or xenogenic host species recipients. These rat RPE-suspected tensions have resulted in the rescue of photoreceptors in the RCS rat model and exhibited limited cell survival and rarely formed a complete monolayer [134]. Rejection by the host immune response is one of the major drawbacks of homologous RPE grafts. The immune response is dependent on the healthy, intact cell layers associated with the subretinal space; thus, graft rejection will constantly delay treatments for retina degeneration [135].

### 5.5. Mature Retinal Cells

Mature retinal cells have been isolated and delivered by researchers to rebuild the retinal photoreceptor layer. Researchers have delivered mature retinal cells in the form of photoreceptor sheets, retinal and photoreceptor cell suspensions [82]. The potential development of synapses between the host and transplant neurons was observed after the transplantation procedure. Though, irregular architectures called rosettes were formed together with the shorted and degenerated segments these mature retinal cells also failed to exhibit incorporation. Thus, because of unsuccessful clinical trials and the inability to improve this host–transplant integration, the mature retinal cells have been out of focus unlike other cell types for the last few years.

### 5.6. Adipose Stem Cells (ASCs)

These are mesenchymal stem cells that can differentiate into numerous cell types such as other stem cells. ASCs are derived from adipose tissues rather than bone marrow [134]. The ASCs also aid in autologous transplantation and lower the risk of immune rejection. Haddad et al., 2013 transplanted human ASCs into rat vitreous cavities and observed a successful integration within the eye. Yet, there is still a risk of migrating cells across the blood–retina barrier and towards the non-targeted area. Thus, the optimization of experiments and ASC cell mechanisms is needed to be understood to use these cells for transplantation [135].

## 6. Materials Aspects for Retinal Implants and Prostheses

To ensure that the electronic system can effectively deliver a stimulating current to the retina, the role of material plays an important role. These electronic implants should not significantly damage the surrounding tissue but should work impactfully. Delicate retinal tissue can be affected by inflammation and infection and, hence, any introduced device can cause acute and delayed foreign-body types of immunologic responses. The patients can also develop hypersensitivity reactions to polymers and metals. The substances such as antioxidants, contaminants and oxidants may be left as residues on implants and can cause inflammation [136].

The main components used for retinal implants are the hermetic case, the bond pads, the conducting feed throughs, the electrodes, the conductive leads, and the flexible polymer substrates. The mechanical design of the intraocular electrode array is considered important regarding the safety of electrical stimulation. The retina is extremely delicate and about 200 μm thick with a Young’s modulus of only 20 to 40 kPa [137], optimized and designed in the thin form of polymer arrays and to achieve safe implantation, materials used for retinal implants and protheses are PDMS, polyimide and parylene. 

Polydimethylsiloxane (PDMS) is a group of polymeric organosilicon compounds, referred to as silicones that have well-known rheological properties. It has a very low transition temperature Tg ~ 125 °C, unique flexibility characteristics, with a shear elastic modulus G ~ 250 kPa. These factors, plus the ease with which it can be used in a cleanroom setting, make PDMS extremely applicable for incorporation into micro-electronic electrode arrays required for the retinal prosthesis [138].

The commercially available thermosetting polyimides are available in multiple forms, such as resins, sheets and machine parts which are currently being used for microelectronic circuits as dielectrics because of their higher signal transmission speed. Polyimides while maintaining mechanical strength, thermal stability and chemical resistance, can achieve a low dielectric constant [139].

Another class of polymer used in medical retinal prothesis is parylene. It is the generic name used for a variety of chemical vapor-deposited poly (p-xylylene) polymers. Parylene can be produced ultrathin, uniform, conformal and pinhole-free by using vapor deposition polymerization. It has a low permeability, high tensile strength, moisture and dielectric barrier properties. Among the most commonly used parylenes is parylene C, it is inert, clear, hydrophobic and biocompatible [140].

All these polymers are capable of being molded into an array and can be metallized, designs that conform to the curvature of the retina. Although, both polyimide and parylene when produced as thin sheets can cut the retina. This is why coating of the parylene or polyimide is required with silicone for retinal prosthesis, which is a much softer polymer. In a recent study on the biocompatibility of materials used in subretinal prostheses, the poly (ethylene glycol) PEG, parylene and poly (vinyl pyrrolidone) PVP produced less histological disruption than polyimide or polyimide coated with either amorphous aluminum oxide or amorphous carbon [141].

## 7. Summary and Perspectives

Present retinal tissue engineering strategies, which combine both cells with substrate materials, initiated two decades ago with the assessment of various cell cultures on different nanostructured materials. Researchers have shown that a wide choice of natural, synthetic, as well as hybrid nano-substrates, can be occupied with different types of cells such as iPSC, adipose stem cells, mature retinal cells, etc. for successful retinal transplantation therapies. The development of such a construct can restore vision to patients with advanced RPE and photoreceptor loss with successful tissue-engineered therapy. Cell replacement is likely to become a major implementation in the forthcoming progress of regenerative medicine with the possibility of endogenous regeneration. The delivery and integration of regenerative nanostructured scaffolds to the subretinal space of the eye should overcome the chances of immune rejection, functional neural growth to form efficient connections. These nano-constructs may help to control several challenges by improving the cell integration, delivery and existence of transplanted cells. In the future, more clinical and pre-clinical trials are needed that help to decide the best formulation scheme.

For more than two centuries, restoring functional vision using electrical stimulation has been a goal of ophthalmologists and vision scientists. To aid the blind in regaining some degree of vision, significant progress has been made towards the development of prostheses. The first human trials have provided encouraging results for the use of electrically active retinal prostheses and micro photodiode arrays. Although the substantial benefits can be obtained in clinical trials specifically in improved mobility and localization of the object. Patients are generally pleased by using implants and devices in their everyday lives. However, the need for improvement still exists. The patients do not report “pixelized vision while using the prosthesis in the real world,” where each electrode creates a spot of light and creating the form of vision is a major challenge. The patients with an implant cannot yet recognize the details of faces or objects and image fading also must be addressed, since patients need to rely on the quality of images throughout the day. The resolution of implants must be increased, by increasing both the density of electrodes and the number of electrodes to achieve this important goal. Moreover, to date, the integration of technology with the brain is the topmost challenge [142,143].

## 8. Method of Literature Search

For this review, the researchers conducted a MEDLINE/PubMed search for articles published till 2021, using the following keywords: “nanofibers”, “nanostructured”, “retinal degenerative disease”, “nano biomaterials and retina”, “polymers and retinal regeneration”, “cell therapy for retinal regeneration”, “retinal implants” and “retinal prosthesis”. Current contents and relevant articles on the role of nanomaterials and cell therapy in retinal regeneration were also obtained using a Google search. Published papers in languages other than English were excluded. We read all 143 articles and did not contact any authors.

## Figures and Tables

**Table 1:** Summary selected biomaterials from the biocompatibility and medicinal use point of view.

Type	Forms and Thickness	Advantages	Disadvantages	Applications
Natural biomaterials				
Gelatin	30–70 μm film	high integration with host cells	retraction of photoreceptor axons	in vivo [21,22,25] ex vivo [23,26]
Collagen	thin film (7 μm)	support of integration in the host tissue, upregulation of angiogenesis	Ill-defined degradation time, low effectivity and porosity of thin films in comparison with nanofiber scaffolds	in vivo [30,31,32] ex vivo [33]
Fibrinogen	thin film	stimulation of proliferation and differentiation of transplanted cells	short degradation time, gentle local inflammatory response	in vivo [35,36] in vitro [37]
Laminin	thin film	promotion of the cell adhesion	short degradation time	in vivo [39,40]
Synthetic biomaterials				
Hydrogel	various forms and thickness, mainly very thick	minimally invasive (injectable)	unsuitable for cell culturing, transplantation of epithelial monolayer	in vivo [16]
Poly (lactic-co-glycolic acid) (PLGA) and Poly (L-lactide-co-DL-lactide) (PDLLA)	1–100 μm films, nanofibers, microspheres	the very high porosity of nanofibrous membrane, stimulation of proliferation and differentiation of transplanted cells, support of the cell adhesion	nanofibrous membranes with thickness below 10 μm require the supporting ring	in vivo [5,45,46] ex vivo [46,47,49]
Poly(methyl methacrylate) (PMMA)	thin films 6 μm	support of integration	non-biodegradable	in vivo [50] in vitro [64]
Polyglycerol sebacate (PGS)	thin films 45 μm	mechanical properties (very soft and elastic), biodegradability, high levels of cell survival	require the use of a thick layer for maintaining of plain shape	in vivo [52,53,55] in vitro [51,54]
Poly-urethanes	thin films	biodegradability, degradation of oxyradicals, high porosity	hydrophobic surface with poor cell adherence	in vivo [58,59] in vitro [57]
Thermo-responsive polymer	liquid form	support of the formation of RPE monolayer	require the backbone (of collagen)	in vivo [60,63]
Poly (e-caprolactone) (PCL)	thin films from 200 nm to 5 μm	biodegradability, high permeability	very slow degradation (longer than 3 years), acidic degradation products	in vivo [63,65,66,67] in vitro [64,69,70,71,72]

## Data Availability

Data available in a publicly accessible repository.
